# A Defined and Xeno-Free Culture Method Enabling the Establishment of Clinical-Grade Human Embryonic, Induced Pluripotent and Adipose Stem Cells

**DOI:** 10.1371/journal.pone.0010246

**Published:** 2010-04-19

**Authors:** Kristiina Rajala, Bettina Lindroos, Samer M. Hussein, Riikka S. Lappalainen, Mari Pekkanen-Mattila, Jose Inzunza, Björn Rozell, Susanna Miettinen, Susanna Narkilahti, Erja Kerkelä, Katriina Aalto-Setälä, Timo Otonkoski, Riitta Suuronen, Outi Hovatta, Heli Skottman

**Affiliations:** 1 Regea - Institute for Regenerative Medicine, University of Tampere, Tampere University Hospital, Tampere, Finland; 2 Biomedicum Stem Cell Center and Children's Hospital, University of Helsinki, Helsinki, Finland; 3 Division of Medical Nutrition, Department of Biosciences and Nutrition, Karolinska Institutet, Stockholm, Sweden; 4 Department of Laboratory Medicine, Karolinska Institutet, Karolinska University Hospital, Stockholm, Sweden; 5 Department of Biomedical Engineering, Tampere University of Technology, Tampere, Finland; 6 Department of Eye, Ear and Oral Diseases, Tampere University Hospital, Tampere, Finland; 7 Department of Clinical Science, Intervention and Technology, Karolinska Institutet, Karolinska University Hospital, Stockholm, Sweden; University of São Paulo, Brazil

## Abstract

**Background:**

The growth of stem cells in *in vitro* conditions requires optimal balance between signals mediating cell survival, proliferation, and self-renewal. For clinical application of stem cells, the use of completely defined conditions and elimination of all animal-derived materials from the establishment, culture, and differentiation processes is desirable.

**Methodology/Principal Findings:**

Here, we report the development of a fully defined xeno-free medium (RegES), capable of supporting the expansion of human embryonic stem cells (hESC), induced pluripotent stem cells (iPSC) and adipose stem cells (ASC). We describe the use of the xeno-free medium in the derivation and long-term (>80 passages) culture of three pluripotent karyotypically normal hESC lines: Regea 06/015, Regea 07/046, and Regea 08/013. Cardiomyocytes and neural cells differentiated from these cells exhibit features characteristic to these cell types. The same formulation of the xeno-free medium is capable of supporting the undifferentiated growth of iPSCs on human feeder cells. The characteristics of the pluripotent hESC and iPSC lines are comparable to lines derived and cultured in standard undefined culture conditions. In the culture of ASCs, the xeno-free medium provided significantly higher proliferation rates than ASCs cultured in medium containing allogeneic human serum (HS), while maintaining the differentiation potential and characteristic surface marker expression profile of ASCs, although significant differences in the surface marker expression of ASCs cultured in HS and RegES media were revealed.

**Conclusion/Significance:**

Our results demonstrate that human ESCs, iPSCs and ASCs can be maintained in the same defined xeno-free medium formulation for a prolonged period of time while maintaining their characteristics, demonstrating the applicability of the simplified xeno-free medium formulation for the production of clinical-grade stem cells. The basic xeno-free formulation described herein has the potential to be further optimized for specific applications relating to establishment, expansion and differentiation of various stem cell types.

## Introduction

Stem cells are invaluable tools for research, drug screening, to study diseases and can potentially serve as a resource for regenerative therapies. Multipotent adipose stem cells (ASCs) exhibiting immunoprivileged properties are an attractive and abundant stem cell source for regenerative medicine that upon induction can undergo adipogenic, osteogenic, chondrogenic, neurogenic and myogenic differentiation *in vitro* [1]–[3]. However, even more expectations on clinical applicability in diverse fields of cell- and tissue-replacement therapies are focused on pluripotent stem cells. Besides of hESCs, a promising new source of pluripotent cells was recently discovered, as human somatic cells were reprogrammed by introducing a set of transcription factors linked to pluripotency to yield induced pluripotent stem cells (iPSC) [4], [5]. Human iPSCs are a potential source of patient-specific pluripotent stem cells that could be used to treat a number of human degenerative diseases without evoking immune rejection. From these stem cell types, only patient specific ASCs have so far been used in clinical cell therapy while clinical trials using hESCs is at the very beginning (http://www.geron.com). Many major challenges including teratoma formation, immunogenicity and the use of oncogenes and retroviruses in the reprogramming of iPSCs need to be addressed before hESCs and iPSCs can be safely used as a source for clinical cell therapy.

One of the major challenges for the clinical use of stem cells is the exposure to undefined animal-derived products during *in vitro* establishment and expansion of the cells. Considerable progress has been made towards the generation of defined culture conditions for stem cells. FBS has been mostly replaced with knockout- serum replacement (KO-SR, Invitrogen) [6]–[8] and human feeder cells have been successfully used to replace mouse embryonic fibroblasts (MEFs) in the derivation and expansion of hESCs and iPSCs [9]–[15]. In addition, various feeder cell-free culture conditions have been developed for the culture of hESCs and iPSCs [16]–[19]. Despite the progress, most existing stem cell lines have been exposed to a variety of undefined animal-derived products which makes these cell lines undesirable for clinical applications. In addition to establishment and culture of stem cells, many differentiation protocols utilize a variety of undefined products that may have unknown effects to the cell characteristics and differentiation. The potential consequences of transplanting human cells exposed to animal-derived products into patients include an increased risk of graft rejection, immunoreactions, and viral or bacterial infections, prions, and yet unidentified zoonoses [20]–[22]. Therefore, optimization and standardization of a fully defined xeno-free establishment, culture and differentiation methods for stem cells is needed for research and especially for clinical application.

Since there is a clear indication that hESCs cultured without feeder cells in long-term cultures may be more prone to undesirable abnormalities caused by suboptimal culture conditions and enzymatic passaging of hESCs [23], [24], we believe that the goal for developing clinical-grade hESCs and iPSCs should be focused on the use of qualified human feeder cells. In the present study we have developed a completely new defined xeno-free medium capable of supporting the expansion of hESCs, iPSCs and ASCs while maintaining their characteristics. In addition, we describe the derivation and long-term culture of new hESC lines using the xeno-free medium.

## Results

### Initial development of a xeno-free culture medium formulation

We developed a xeno-free medium (RegES) composed of a knockout-Dulbecco's modified Eagle's medium (KO-DMEM, Invitrogen, Carlsbad, CA) base supplemented with human serum albumin, amino acids, vitamins, antioxidants, trace minerals, and growth factors (Table S1). All medium components were synthetic, recombinant, or of human origin. Removal of any of the components resulted in reduced growth or excess differentiation of hESCs (data not shown). Initially, the xeno-free RegES medium was evaluated with hESC lines HS237, HS346 and HS401 [25], [26] and it was noticed that the colonies were thinner and the growth of the hESCs was slower than in hESC lines derived and cultured in the conventional medium containing KO-SR (hES medium, data not shown). Despite the thinner colonies and slower growth, new hESC line, Regea 06/015, was successfully derived using the first version of the xeno-free RegES medium and hFF cultured in human serum. After a couple of passages, the growth of the Regea 06/015 cell line declined and, to avoid losing the cell line, the cell line was transferred at passage 7 to conventional culture medium containing KO-SR. Since then, we have been culturing the Regea 06/015 cell line continuously for over 3 years in hES medium containing KO-SR. The cell line exhibits normal hESC characteristics including pluripotency as determined by embryoid body analysis and teratoma formation and is karyotypically normal (Figure S1, S2, S3).

**Figure 1:**
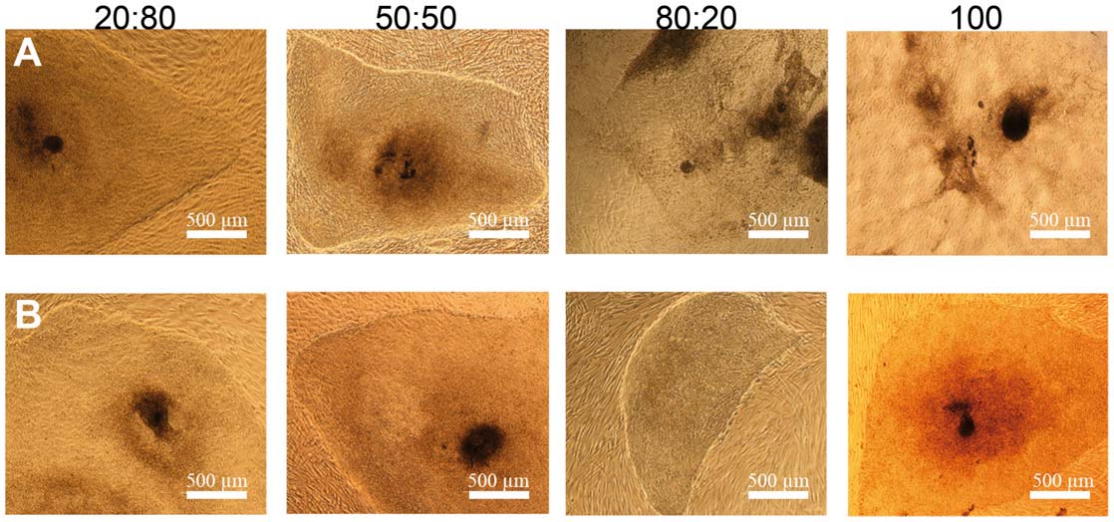
Comparison of RegES and HEScGRO xeno-free culture media. Human ESC line HS401 during the adaptation phases in RegES culture medium and in HEScGRO medium. Human ESC line HS401 was transferred from hES culture medium to new culture conditions using stepwise adaptation: 20∶80, 50∶50, 80∶20 and 100%. **A**) Adaptation phases to HEScGRO medium. Note excess differentiation of hESC colonies at adaptation phases 80∶20 and after completion of the adaptation (100%). **B**) Adaptation phases to RegES culture medium.

Next, the medium was further optimized by addition of several different components and by adjusting the osmolarity to 320–330 mOsm/kg. (Table S1). These improvements markedly enhanced the cell characteristics, including the colony morphology and growth (data not shown). Comparison of xeno-free RegES medium with the commercially available xeno-free HEScGRO medium specifically developed for hESC cultures using feeder cells revealed that in our study and in contrast to RegES, HEScGRO medium was not able to maintain the undifferentiated growth of the hESCs but instead resulted in excess differentiation as judged by the morphology of the hESC colonies (Fig. 1). For that reason in the following studies, the hESC line Regea 06/040 derived and cultured in the hES medium was used as a control cell line instead of hESC line cultured in HEScGRO.

### Optimized formulation supports the derivation and culture of new hESC lines

**Figure 2:**
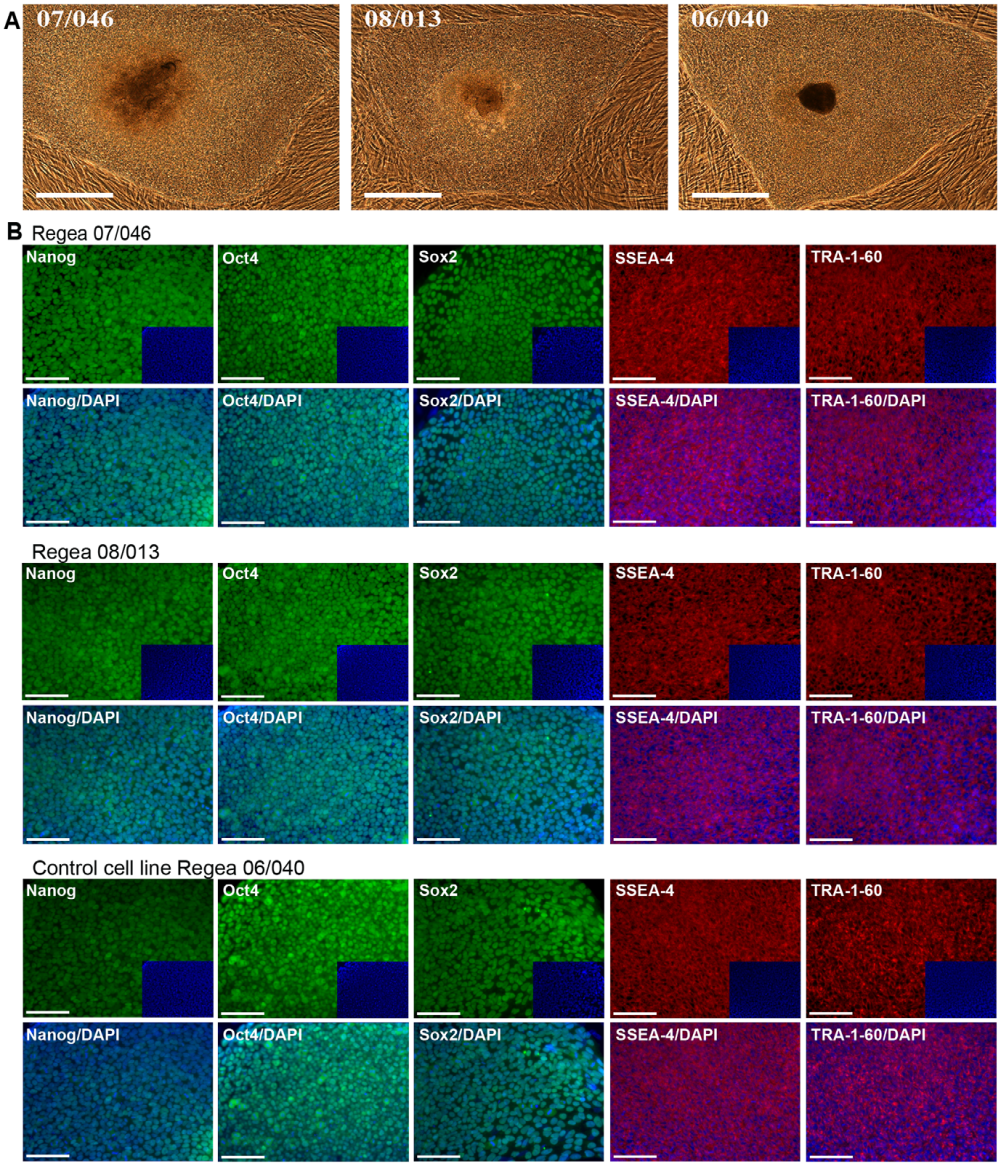
Morphology and immunocytochemical characterization of hESC lines. **A**) Bright-field (scale bar, 500 µm) microscopic images showing undifferentiated colony morphology of hESC lines Regea 07/046 at passage 44, Regea 08/013 at passage 12, and control cell line Regea 06/040 at passage 45. **B**) Fluorescent images (scale bar, 100 µm) showing qualitative immunocytochemistry of cells positive for Nanog, Oct4, Sox2, SSEA-4 and TRA-1-60. Insets represent DAPI staining. Regea 07/046 at passage 63, Regea 08/013 at passage 51, and control cell line Regea 06/040 at passage 42.

**Figure 3:**
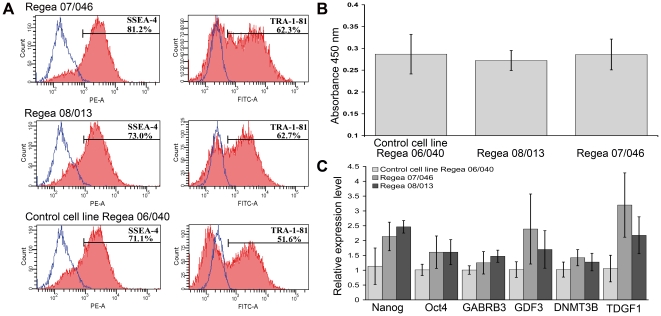
Stem cell marker expression and proliferation of hESC lines. **A**) Quantitative flow cytometry analyses showing comparable expression levels of SSEA-4 and TRA-1-81 in all hESC lines at day 7. Regea 07/046 at passage 45, Regea 08/013 at passage 41, and Regea 06/040 at passage 26. **B**) Cell proliferation analysis demonstrating comparable growth rates of hESC lines Regea 06/040 at passage 29, Regea 07/046 at passage 53 and Regea 08/013 at passage 41. **C**) Quantitative RT-PCR analysis of *Nanog*, *Oct4*, *GABRB3*, *GDF3*, *DNMT3B* and *TDGF1* expression in hESC lines Regea 07/046 at passage 52, Regea 08/013 at passage 45, and Regea 06/040 at passage 33. No significant differences (over 2-fold) were detected between hESC lines.

**Figure 4:**
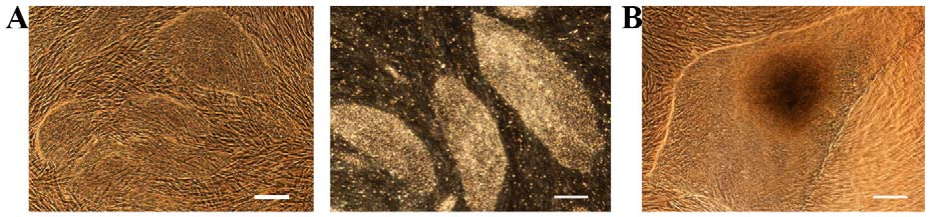
Enzymatic passaging, freezing and thawing of hESC in RegES medium. **A**) Bright-field (scale bar, 500 µm) microscopic images showing undifferentiated colony morphology of hESC line 08/013 (p40) cultured in RegES medium and passaged with a single-cell dissociation method for 6 passages. **B**) Bright-field (scale bar, 500 µm) microscopic image showing undifferentiated colony morphology of hESC line 07/046 (p33) after freezing and subsequent thawing in RegES medium at passage 1.

The optimized RegES medium was used to derive two additional hESC lines (Regea 07/046 and Regea 08/013; Fig. 2 and 3) from 26 surplus embryos that did not undergo proper blastocyst formation. Both hESC lines have been continuously cultured for over 80 passages. The cell lines have been maintained by mechanical passaging but a single-cell enzymatic dissociation (SCED) method developed and described by Ellerstöm and co-workers [27] utilizing recombinant enzyme TrypLE Select (Invitrogen), is feasible with RegES as well (Fig. 4A). With the enzymatic propagation method, hESCs can be passaged with a 1∶10 split ratio, enabling a rapid increase in the number of cells. In addition, the RegES medium can also be used for freezing and thawing of the hESCs (Fig. 4B).

Both of the hESC lines derived and cultured in RegES medium have been karyotyped regularly and exhibit a normal diploid karyotype (Figure S2). Analysis of these cell lines by immunocytochemical stainings (Nanog, Oct4, Sox2, SSEA-4 and TRA-1-60) and flow cytometry (SSEA-4, TRA-1-81) demonstrated that both cell lines express markers that are characteristic of hESCs at levels comparable to the control hESC line Regea 06/040 derived and cultured using hES medium (Fig. 2B, 3A). In quantitative reverse transcription PCR (qRT-PCR) no significant differences (over 2-fold) in the expression levels of markers *Nanog*, *Oct4*, *GABRB3*, *GDF3*, *DNMT3B* and *TDGF1* were detected between hESC lines derived in RegES and in control hESC line derived in hES medium (Fig. 3C). An enzyme-linked immunosorbent assay (ELISA)-based analysis of cell proliferation showed that the cell proliferation rates of Regea 07/046 (A = 0.286) and Regea 08/013 (A = 0.272) were comparable to that of control cell line Regea 06/040 (A = 0.287) cultured in hES medium (Fig. 3B).

### Human ESCs cultured for prolonged periods in xeno-free culture medium maintain their pluripotency and differentiation characteristics

**Figure 5:**
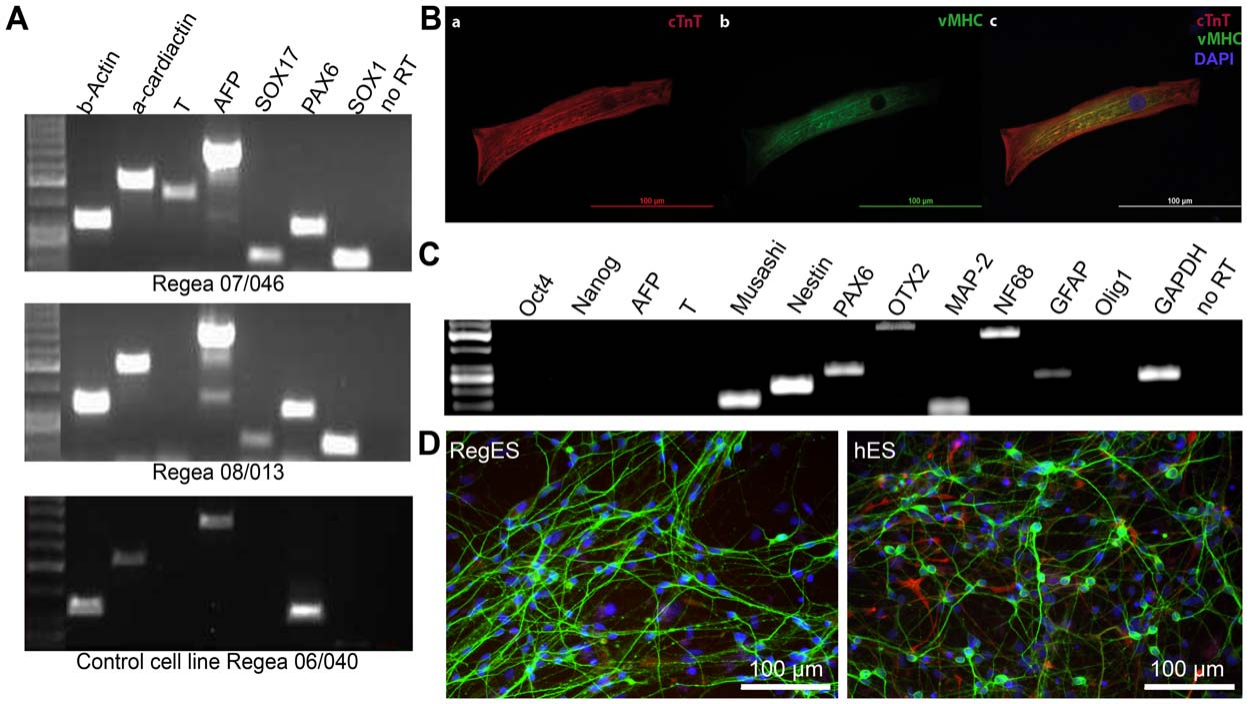
Analysis of pluripotency and differentiation characteristics of the hESC lines derived in xeno-free medium RegES. **A**) RT-PCR analysis of *in vitro*-derived EBs showing transcripts for *AFP* and *SOX17* (endodermal markers), *α-cardiac actin* and *T* (Brachyury; mesodermal markers), *SOX1* and *PAX6* (ectodermal markers), and *β-actin*. Lane 1, 50-bp DNA ladder. Regea 07/046 at passage 42, Regea 08/013 at passage 35, and Regea 06/040 at passage 101. **B**) Differentiated cardiomyocytes from hESC line Regea 08/013 were stained with a) cardiac troponin T (red) and b) ventricular myosin heavy chain (green). c) Merged picture of a) and b) with DAPI staining. Scale bar is 100 µm. **C**) RT-PCR analysis of neurospheres derived from hESC line Regea 08/013 cultured in RegES medium showed expression of neural precursor markers *Musashi*, *Nestin* and *PAX6*; neuronal markers *MAP-2*, *NF68* and *OTX2*; and astrocytic marker *GFAP*. No expression of pluripotent markers *Oct4* and *Nanog*, nor endo- *AFP* or mesodermal markers *T/Brachyury* were detected. **D**) Most of the cells migrating out from the plated neurospheres stained positive for neuronal marker MAP-2 (green) and few cells were positive for astrocytic marker GFAP (red). DAPI staining (blue). Scale bar is 100 µm.

To confirm that the new cell lines maintain their pluripotency *in vitro*, we performed an embryoid body (EB) assay. The EB-derived cells from the cell lines Regea 07/046 and 08/013 as well as from the control cell line Regea 06/040 expressed markers from the three different embryonic lineages; endoderm, ectoderm, and mesoderm, although the expression of *T* was not detected in cells from Regea 08/013 cell line, and *T*, *SOX17* and *SOX1* were not detected in cells from control Regea 06/040 cell line (Fig. 5A). As demonstrated for the hESC line 06/015 (Figure S3), an *in vivo* pluripotency assay was also performed for the cell lines Regea 07/046 and Regea 08/013 and for the control cell line Regea 06/040 using teratoma formation in severe combined immunodeficient (scid)-beige mice and structures from all three germ layers were detected (Figure S4, S5, S6).

We also tested whether hESCs derived and cultured in xeno-free conditions can differentiate to cardiomyocytes and neural cell lineages. To initiate cardiomyocyte differentiation, undifferentiated hESC colonies were dissected into aggregates and plated on the top of END-2 cells [28]. Spontaneously beating areas were observed after 12-16 days after the initiation of the cardiac differentiation. Dissociated, spontaneously beating cells had striated patterning and were stained with cardiac troponin T and ventricular myosin heavy chain markers (Fig. 5B). To generate neuronal cells, hESC colonies cultured in RegES and hES media were dissected into small clusters and cultured in suspension for up to 20 weeks [29]. The differentiated cells expressed neural precursor markers, neuronal markers and astrocytic marker in RT-PCR (Fig. 5C). Immunocytochemical staining verified the neuronal and glial fate of the cells (Fig. 5D). The cardiac and neural differentiation efficiency was similar in hES and RegES medium (data not shown). These results indicated that hESC lines derived and cultured in xeno-free culture medium maintain their pluripotency and furthermore cardiomyocytes and neuronal cells can be generated from these cell lines.

### Xeno-free conditions support the culture of human iPSCs

**Figure 6:**
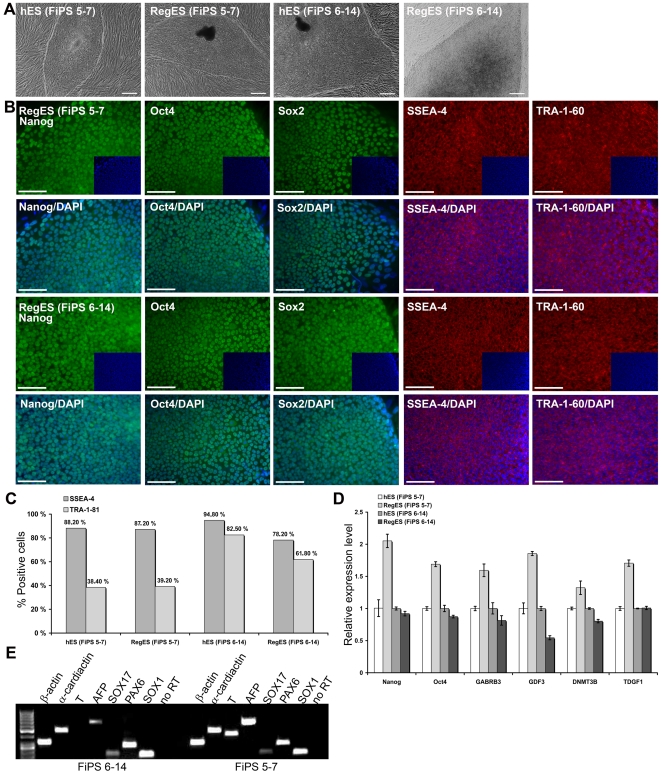
Characterization of iPSC lines. **A**) Bright-field microscopic images (scale bar, 500 µm) showing undifferentiated colony morphology of iPSCs cultured in hES and RegES media. Cell line FiPS 5-7 in hES medium is at passage 15, in RegES medium at passage 5 and FiPS 6-14 in hES medium at passage 18, in RegES medium at passage 9. **B**) Fluorescent microscopic images (scale bar, 100 µm) of iPSCs cultured in RegES medium showing cells positive for Nanog, Oct4, Sox2, SSEA-4 and TRA-1-60. Insets represent DAPI staining. Cell line FiPS 5-7 is at passage 23 and FiPS 6-14 at passage 26. **C**) Quantitative flow cytometry analyses indicating expression of SSEA-4 and TRA-1-81 of iPSC lines cultured in hES and RegES media. Cell samples cultured in hES medium are from 6 day old colonies, cell samples from cell line FiPS 5-7 cultured in RegES from 7 day old colonies and samples from cell line FiPS 6-14 from 8 day old colonies. Cell line FiPS 5-7 in hES medium at passage 15, in RegES medium at passage 14 and FiPS 6-14 in hES medium at passage 16, in RegES medium at passage 7. **D**) Quantitative RT-PCR analysis of *Nanog*, *Oct4*, *GABRB3*, *GDF3*, *DNMT3B* and *TDGF1* expression of day 6 colonies in iPSC lines FiPS 5-7 in hES medium at passage 10, in RegES medium at passage 7 and FiPS 6-14 in hES medium at passage 11, in RegES medium at passage 8. No significant differences (over 2-fold) were detected between iPSC lines. **E**) RT-PCR analysis of *in vitro*-derived EBs showing transcripts for *AFP* and *SOX17* (endodermal markers), *α-cardiac actin* and *T* (Brachyury; mesodermal markers), *SOX1* and *PAX6* (ectodermal markers), and *β-actin*. Lane 1, 50-bp DNA ladder. Both cell lines at passage 10.

To further demonstrate the performance of the developed medium for human pluripotent cells, we cultured two iPSC lines on human feeder cells in these conditions. Currently, both iPSC lines have been continuously cultured in RegES medium for over 20 passages. The morphology and molecular characteristics of the cells are similar as compared to the cells cultured in hES medium as demonstrated by immunocytochemical stainings (Nanog, Oct4, Sox2, SSEA-4 and TRA-1-60), flow cytometry analysis (SSEA-4, TRA-1-81) and qRT-PCR were no significant differences (over 2-fold) in the expression levels of markers *Nanog*, *Oct4*, *GABRB3*, *GDF3*, *DNMT3B* and *TDGF1* were detected between iPSC lines cultured in RegES and in hES medium (Fig. 6A-D). In addition, analysis of EBs demonstrated that iPSCs cultured in xeno-free RegES medium maintain their ability to differentiate to all three germ layers although the expression of *T* was not detected in cells from FiPS 6–14 cell line (Fig. 6E).

### Xeno-free conditions enhance proliferation and cell surface marker expression profile of human ASCs

**Figure 7:**
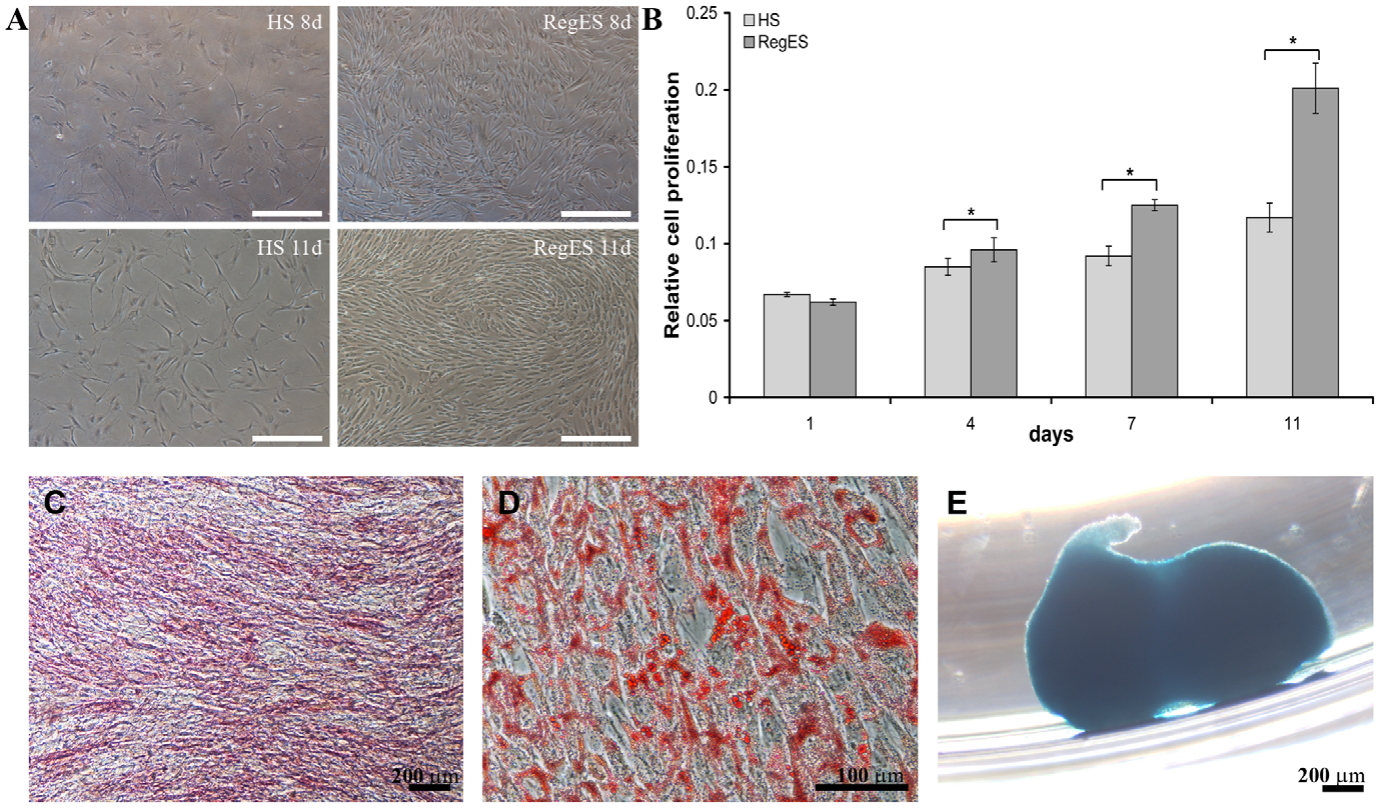
Characterization of human ASCs. **A**) Morphology of ASCs cultured in HS medium and RegES medium at day 8 and day 11. (Scale bar 500 µm) **B**) WST-1 proliferation assay. Proliferation of ASCs was examined in HS medium and RegES medium analyzed at time points 1, 4, 7, and 11 days. The data in diagram is presented as mean ± SD. *p<0.05 (n = 7 donors with 4 replicate wells). Significantly higher proliferation rates were detected in ASCs cultured in RegES medium at time points 4, 7 and 11 days when compared to ASCs cultured in HS medium. **C**) Osteogenic differentiation of ASCs at passage 3 in RegES medium demonstrated by alkaline phosphatase staining 14 days after the initiation of the differentiation. **D**) Adipogenic differentiation of ASCs at passage 3 in RegES medium confirmed by Oil red O staining after 14 days of differentiation. **E**) Chondrogenic differentiation of ASCs at passage 3 in RegES medium after 14 days of differentiation as verified by Alcian blue staining.

ASCs isolated from adipose tissue samples were used to assess the performance of xeno-free RegES medium for the culture of mesenchymal stem cells. To determine the proliferation rate of ASCs grown in xeno-free RegES medium and allogeneic human serum-containing medium (HS medium) we performed the WST-1 proliferation analysis at several time points (1, 4, 7 and 11 days). Seven ASC lines were used for the analysis in both conditions. Concurrently, cell morphology was observed by light microscopic examination to confirm the proliferation assay results (Fig. 7A). The proliferation analysis showed already at day 4 that cultures with RegES medium exhibited a higher proliferation rates of ASCs as compared to HS medium (Figure 7B). Subsequently, ASCs continued to proliferate at a higher rate in RegES medium compared to HS medium at day 7 and 11. Significant differences in the proliferation rates were observed between the RegES medium and HS medium at day 4 (p = 0.035), day 7 (p = 0.022) and day 11 (p = 0.018) (Fig. 7B).

Flow cytometric characterization was performed to compare surface marker expression characteristics of ASCs expanded in xeno-free RegES medium and HS medium (Table 1). Four cell lines were analyzed for every culture condition. The flow cytometry analysis revealed >50% expression for ASCs cultured in both culture conditions for the adhesion molecule CD105 and extracellular matrix protein CD90. ASCs cultured in both culture conditions showed no expression (<4%) of CD34 and CD45, markers of hematopoietic cell lineage. Additionally, no expression (<1%) of MHC Class II isotype HLA-DR was observed for ASCs cultured in HS or RegES media and modest expression (10%) of MHC Class I isotype HLA-ABC was seen in RegES cultured ASCs. While both culture conditions maintained the characteristic surface marker expression profile of ASCs, statistical analysis revealed significant differences in the expression of sialomucin-like adhesion molecule CD34 (p = 0.043), leucocyte common antigen CD45 (p = 0.017), adhesion molecule CD105 (p = 0.020) and MHC Class I isotype HLA-ABC (p = 0.021) of ASCs cultured in HS and RegES media.

**Table 1:** Surface marker expression characteristics of ASCs cultured in HS and RegES media.

Surface Protein	Antigen	HS medium (n = 4)	RegES medium (n = 4)
CD34 *	Sialomucin-like adhesion molecule	3.5%±1.7	1.2%±0.7
CD45 *	Leukocyte common antigen	0.4%±0.0	2.4%±1.2
CD90	Thy-1, T-cell surface glycoprotein	93.1%±11.2	99.8%±0.1
CD105 *	SH-2, endoglin	52.0%±8.3	75.7%±6.6
HLA-ABC *	Major histocompatibility class I antigen	0.6%±0.4	10.0%±11.4
HLA-DR	Major histocompatibility class II antigen	0.8%±0.6	0.4%±0.1

Cell lines 5/08, 19/08, 24/08 and 25/08 cultured in HS medium were at passage 2–3 and cell lines 9/08, 11/08, 25/08 and 31/08 cultured in RegES medium were at passage 3–4. Data are presented as mean ± standard deviation from the number of donors/samples indicated in parentheses.

* p<0.05.

The multilineage differentiation capability of ASCs expanded in RegES was examined by culturing cells at passage 3 under conditions that support osteogenic, adipogenic and chondrogenic differentiation. The osteogenic induction was evident after 14 days in culture in osteogenic RegES medium (Figure 7C). After less than 7 days of adipogenic induction, accumulation of small lipid droplets was visible in the cells grown in adipogenic RegES medium by light microscopic inspection within the ASCs (data not shown). At day 14, ASCs in RegES adipogenic induction cultures proliferated rapidly and grew in a layered manner, with lipid droplets forming in several layers (Figure 7D). In the chondrogenic induction cultures, ASCs expanded in chondrogenic RegES medium began to aggregate and form condensed pellets within 2–3 days after induction. At day 14, the chondrogenic induction was confirmed with Alcian blue staining (Figure 7E).

## Discussion

As the number of potential applications for stem cell transplantation in the treatment of various degenerative diseases is rapidly increasing a strong focus is needed on safety, reproducibility and quality of stem cell transplants. To generate therapeutically safe and usable stem cell-derived products for clinical cell therapies, all animal-derived material must be eliminated from the establishment, culture, and differentiation steps. A completely xeno-free culture system was described by Ellerström and co-workers [30] in which they used a xeno-free derivation procedure, human serum, and xeno-free human foreskin feeder (hFF) cells to derive a hESC line. We have previously demonstrated, however, that several commercial xeno-free serum replacements and human sera are not suitable for long-term undifferentiated culture of hESCs [31]. Recently Crook and co-workers described the proof-of-concept for the generation of six clinical-grade hESC lines using hFF as feeder cells [32]. These lines were established and banked in a clinical-grade manufacturing process (cGMP) facility in compliance with international regulatory requirements. Although they used cGMP-compliant reagents and materials in the production process of the hESC lines, not all the reagents were xeno-free, possibly compromising the safety of the established hESC lines for clinical application. In this study, we have identified all the essential nutrients and growth factors required to enable feasible growth characteristics and to maintain the undifferentiated state of hESCs, iPSCs and ASCs. The described RegES medium formulation is designed to meet the special requirements of stem cell culture and the quality requirements for clinical use of stem cells.

Our data demonstrate that hESCs can be successfully derived and cultured long-term in the RegES formulation while maintaining their undifferentiated state. Furthermore, EB formation, teratoma analysis and differentiation of cardiomyocytes and neural cells demonstrated that hESCs derived and cultured in xeno-free RegES medium maintain their pluripotency and differentiation characteristics. The characteristics and differentiation potential of hESCs cultured in the xeno-free RegES medium were comparable to hESCs cultured in the conventional hES culture medium. In the comparison of the RegES medium to the xeno-free commercially available HEScGRO medium it was noticed that HEScGRO was unable to maintain the undifferentiated growth of hESCs. Human ESCs can be frozen and thawed using the xeno-free medium RegES and both mechanical passaging and single-cell enzymatic dissociation methods can be used. With enzymatic single-cell propagation, the number of cells can be multiplied rapidly, thereby enabling up-scaling and large-scale cultivation of hESCs [27]. As demonstrated with the first derived hESC line Regea 06/015, hFF can be cultured without any animal-derived materials, enabling the establishment and production of xeno-free hESC lines in the future. Furthermore, our formulation enables feasible culture environment for iPSCs. We believe that these results suggest that the derivation of iPSC lines is also possible by using xeno-free RegES medium and should be tested.

In the culture of ASCs the replacement of FBS with pooled allogeneic human serum and human serum derivatives has been reported to support equal or higher proliferation rates and multipotentiality [33]–[35]. However, serum composition is largely unknown, and shows significant lot-to-lot variability that may affect the reproducibility of the results [2]. In clinical therapy, a feasible option would be to use patient's autologous serum but it may not always be available in large quantities. In the culture of ASCs our results show that the xeno-free RegES medium exhibited significantly higher proliferation rates compared to the reference media containing HS. Both culture conditions maintained the characteristic surface marker expression profile of ASCs with expression of markers verifying the mesenchymal origin of cells and no expression of markers of the hematopoietic origin of cells. However, statistical analysis revealed significant differences in the surface marker expression of ASCs cultured in HS and RegES media. In addition, RegES culture medium is able to maintain the differentiation capacity of the ASCs and thus maintain the multipotency of the ASC population. Our results indicate that RegES medium possess all the inherent promise for translational use in clinical applications and after additional pre-clinical safety and efficacy studies, it could be used to replace autologous or allogenous HS in the clinical cell therapy studies with ASCs undergoing in our institute [3] and elsewhere.

Although all stem cell lines worldwide share the expression of characteristic stem cell markers, different culture conditions are known to influence gene expression and therefore many of the cell properties. The use of completely defined conditions will allow for better understanding of stem cell regulation and provide more reproducible results. Currently, there are at least three commercially available xeno-free culture media, HEScGRO (Millipore), developed for the culture of hESC lines with feeder cells, KnockOut SR Xeno-free (Invitrogen) which can be used with or without feeder cells and TeSR2 (StemCell Technologies) for the culture of hESC lines in feeder-cell free conditions. In the present study we have successfully developed a new totally xeno-free effective culture medium for human ESCs, iPSCs and ASCs. These cells can be maintained in the same defined xeno-free medium formulation supporting feasible proliferation while maintaining their characteristics. In addition, we have described the derivation of new hESC lines using the xeno-free RegES medium and showed that these lines retain their differentiation potential after long-term culture in the xeno-free RegES culture medium. However, further studies of *in vitro* differentiated derivatives are needed to evaluate the functionality of the differentiation protocols with the xeno-free RegES medium. Recently a large-scale multi-laboratory comparison study of different culture conditions for hESCs was published [36]. Future studies should focus on comparison of different xeno-free culture conditions and on validation of these conditions to demonstrate long-term culture, maintenance of key features of self-renewal, pluripotency, and genetic stability, as well as derivation, reprogramming or isolation of new stem cell lines as a full proof-of-principle. As many of the current differentiation protocols utilize a variety of undefined products we suggest that xeno-free RegES medium should be tested as a base for differentiation protocols for stem cells. The use of defined products in the differentiation protocols will facilitate the discovery of molecular mechanisms behind the differentiation to various cell types and reproducibility of differentiation processes. Our results indicate that the basic RegES culture medium is applicable for further optimization of xeno-free establishment, culture and differentiation of stem cells and can ultimately serve as a platform for the production of clinical-grade pluripotent stem cells and their derivatives for safer clinical cell therapy treatments.

## Materials and Methods

### Ethics Statement

The study was conducted in accordance with the Karolinska Institutet Ethics Committee to derive and culture hESCs, with the Ethics Committee of Pirkanmaa Hospital District to culture, characterize and differentiate hESCs derived at Karolinska Institutet (R05051), to derive, culture, characterize and differentiate new hESC lines at Regea (R05116) and with the permission of the National Authority for Medicolegal Affairs to do research with human embryos (Dnro 1426/32/300/05). Donated embryos were received from Tampere University Hospital *in vitro* fertilization (IVF) clinic. An informed consent form was signed by both partners after receiving an oral and written description of the study. The donors did not receive financial compensation. Animal experiments were performed at the animal facilities of Karolinska University Hospital or at Turku Center for Disease Modeling and Department of Physiology, Institute of Biomedicine, University of Turku in accordance with the approval of the institution's Ethics Committee. Adipose stem cells (ASCs) were isolated from adipose tissue samples collected from female donors undergoing elective surgical procedures at the Department of Plastic Surgery, Tampere University Hospital with the permission of the Ethics Committee of Pirkanmaa Hospital District. Human iPS cells were derived and characterized at the University of Helsinki, with the permission of the Ethics Committee of the University of Helsinki.

### Cell Culture

The hESC lines HS237, HS346, and HS401 used in the optimization of the xeno-free formulation RegES were initially derived and characterized at the Karolinska Institutet, Stockholm, Sweden [25], [26]. These cell lines were derived and cultured using conventional hES medium containing KO-DMEM basal medium supplemented with 20% KO-SR, 2 mM Glutamax, 0.1 mM β-mercaptoethanol (all from Invitrogen, Carlsbad, CA), 0.1 mM MEM non-essential amino acids (NEAA), 1% penicillin-streptomycin (both from Cambrex Bio Science, Walkersville, MD) and 8 ng ml/ml recombinant human bFGF (R&D Systems Inc., Minneapolis, MN). Human iPSC lines FiPS 5-7 and FiPS 6-14 were derived and characterized at the University of Helsinki. Briefly, human cDNAs of *Oct4*, *Sox2*, *Nanog*, *Klf4* and *Lin28*, were amplified by direct PCR from hESC cDNA, and cloned into pMXs retroviral vector (Addgene Inc., Cambridge, MA). 293-GPG packaging cells were transfected with each pMXs-cDNA vector separately using Fugene HD (Roche Diagnostics GmbH, Mannheim, Germany). Next day, fresh MEF media was added. Viral supernatant was collected 3 times at days 4, 5, and 6 post-transfection, combined and then filtered. The human neonatal foreskin fibroblast line CCCD-1112Sk (ATCC) was plated on gelatin-coated petri dishes and incubated overnight. Cells were then infected with an equally mixed combination of fresh virus-containing supernatant twice at 24 h intervals. At day 3, infected cells were harvested and re-seeded on a Mitomycin C-treated MEF layer. The following day, the medium was changed to hES medium. After 20 to 30 days post-transduction, ES-like colonies were picked, expanded, and characterized as previously described for hESCs [37]. Transgene expression was studied using RT-PCR to confirm transgene silencing. FiPS 5-7 was generated using Oct4, Sox2, Nanog, and Lin28 encoding retroviruses, while FiPS 6-14 was generated using the same factors plus to Klf4.

Human ESCs and iPSCs were gradually adapted to the RegES medium (Table S1) (20∶80, 50∶50, 80∶20, and 100∶0) during the first four passages. Commercially available hFFs (CRL-2429, ATCC, Manassas, VA) cultured in IMDM with L-glutamine and 25 mM HEPES (Invitrogen) supplemented with 10% FBS (Invitrogen) and 1% penicillin-streptomycin (Cambrex Bio Science) were used for hESCs and iPSCs culture. Confluent monolayers of hFFs were mitotically inactivated by irradiation (40 Gy) and plated at a density of 3.8×10^4^ cells/cm^2^. The growth of hESCs and iPSCs was monitored microscopically and culture media were changed daily. The hESC and iPSC cultures were mechanically passaged every 6 to 7 days to new mitotically inactivated feeder cells. Single-cell enzymatic passaging was performed for hESCs as described previously [27] using TrypLE Select (Invitrogen) and an 8 to 12 day splitting interval. The hESCs were frozen using RegES medium supplemented with 10% DMSO (Sigma-Aldrich, St. Louis, MO). Xeno-free commercially available HEScGRO medium (Millipore Corporation, Billerica, MA) without any supplementation was compared to RegES medium for the culture of hESCs.

ASCs were isolated from adipose tissue samples collected from female donors undergoing elective surgical procedures in the Department of Plastic Surgery, Tampere University Hospital. Isolation of ASCs from adipose tissue samples was carried out as described previously [38], [39]. Briefly, the adipose tissue was minced manually into small fragments and digested with 1.5 mg/mL collagenase type I (Life Technologies, Paisley, UK) in a shaking water bath at 37°C. To separate the adipose stem cells from the surrounding tissue the digested tissue was centrifuged and filtered in sequential steps. The isolated cells were expanded in DMEM/F-12 1∶1, supplemented with 1% GlutaMAX, 1% antibiotics/antimycotic (a/a; 100 U/mL penicillin, 0.1 mg/mL streptomycin, and 0.25 µg/mL amphotericin B) (all from Invitrogen) and 10% alloHS (PAA Laboratories GmbH, Pasching, Austria), (HS medium). For testing defined, xeno-free conditions, ASCs were transferred to RegES culture medium in flasks with pre-coated CELLstart (Invitrogen). For detachment of ASCs TrypLE Select (Invitrogen) was used.

### Derivation of hESC lines

Surplus poor quality embryos that could not be used for infertility treatment were obtained as donations from couples undergoing *in vitro* fertilization treatment at Tampere University Hospital, Tampere, Finland. The mechanical derivation of the cell lines from blastocyst-stage embryos with a hardly visible inner cell mass was performed using a specially made flexible metal needle (Hunter Scientific, Essex UK) and surgical knife as described previously [26]. For successful derivation of the 06/015 cell line, we used 10 surplus embryos (arrested around day 3–4) without blastocyst development. Derivation of the cell line Regea 06/015 was performed by removing the zona pellucida and plating the remaining cells on the top of hFF cells using a flexible needle. Human ESC line Regea 06/040 was derived using hES medium and hESC lines Regea 06/015, Regea 07/046, and Regea 08/013 were derived using RegES medium. Human ESC line Regea 06/015 was derived on feeder cells (C-12300 PromoCell GmbH, Heidelberg, Germany) cultured in IMDM with L-glutamine and 25 mM HEPES (Invitrogen) supplemented with 10% human serum (Sigma-Aldrich), 0.1 mM NEAA (Cambrex Bio Science), and 0.1 mM penicillin-streptomycin (Cambrex Bio Science).

### Immunocytochemistry

Immunocytochemistry was performed as previously described [31]. The primary antibodies used for hESCs and iPSCs were specific for Nanog, Oct4 (both from R&D Systems), SSEA-4 and Sox2 (both from Santa Cruz Biotechnology, Inc., Santa Cruz, CA) and TRA-1-60 (Millipore). The cells were incubated with primary antibody solution overnight at 4°C. The cells were probed with Alexa Fluor 568 or Alexa Fluor 488 (both from Invitrogen) secondary antibodies for 1 h in the dark at room temperature. The differentiated cardiomyocytes were incubated overnight at 4°C with anti-cardiac troponin T (Abcam, Cambridge, UK) and anti-ventricular myosin heavy chain (Chemicon). Alexa Fluor 488 or 568 (Invitrogen) conjugated anti-goat and anti-mouse antibodies were used as secondary antibodies. Differentiated neural cells were immunostained after 9 weeks of differentiation. For immunocytochemical staining neurospheres were plated on human laminin (Sigma Aldrich) coated wells in the absence of bFGF and after growing three days the cells were fixed. The fixed neuronal cells were incubated overnight at 4°C with polyclonal rabbit anti- MAP-2 (Chemicon) for neuronal cells and polyclonal sheep anti-GFAP (R&D Systems) for astrocytes. Alexa Fluor 488 and 568 conjugated anti-rabbit or anti-sheep (Invitrogen) were used as secondary antibodies. Human ESCs labeled only with secondary antibodies, were used as negative controls and no fluorescense in these samples were detected. After incubation, the cells were mounted in Vectashield mounting medium containing 4′,6-diamidino-2-phenylindole (Vector Laboratories, Inc., Burlingame, CA). The labeled cells were photographed with an Olympus IX51 phase contrast microscope with fluorescence optics and Olympus DP30BW camera (Olympus Corporation, Tokyo, Japan).

### Alkaline phosphatase staining

The *in vitro* osteogenic differentiation capacity was determined 14 days after the initiation of differentiation by alkaline phosphatase staining (ALP) as described previously [33], [40], [41]. The cell cultures were fixed with a 4% paraformaldehyde (PFA) solution and stained with the leukocyte alkaline phosphatase kit according to the Sigma Procedure No. 86 (#86R-1KT). The ALP staining was confirmed by a purple staining visualized by microscopy.

### Adipogenesis Oil red O staining

The adipogenic differentiation was confirmed with Oil red O staining as described previously [33]. Cell cultures were maintained for 14 days and subsequently fixed with 4% PFA, pretreated with 60% isopropanol and stained with a 0.5% Oil red O solution in 60% isopropanol. After fixation and staining, the wells were rinsed with distilled water and visualized by microscopy. Adipocytes were identified as cells with red-stained lipid vesicles.

### Chondrogenesis Alcian blue staining

The chondrogenic differentiation cultures were maintained for 14 days and were subsequently fixed with 4% PFA, and stained with 1% Alcian blue stain. Cells stained with Alcian blue verified the presence of sulphated proteoglycans within the chondrogenic cell pellet.

### Flow cytometry

Human ESCs and iPSCs cultured in hES and RegES media were analyzed by flow cytometry using antibodies against SSEA-4-PE (R&D Systems) and TRA-1-81-FITC (BD Pharmingen, San Diego, CA). Human ESC samples cultured in hES and RegES media were from 7 day old colonies, iPSC samples cultured in hES medium from 6 day old colonies and samples cultured in RegES medium from 7 day old (5–7) or 8 day old colonies (6–14). Both undifferentiated and differentiated cells present in the culture dish were included in the analyses. Alexa Fluor 488 (Invitrogen) and R-phycoerythrin-conjugated (Caltag-Medsystems Ltd, Buckingham, UK) secondary antibodies were used as isotype controls. ASCs cultured in HS and RegES media (n = 4, passages 2–4) were analyzed by flow cytometry using monoclonal antibodies against CD90-APC (BD Biosciences), CD45-FITC (Miltenyi Biotech, Bergisch Gladbach, Germany), CD34-APC, HLA-ABC-PE, HLA-DR-PE (all from Immunotools GmbH Friesoythe, Germany), and CD105-PE (R&D Systems). The samples were analysed by flow cytometry (FACSAria®, BDBiosciences). The acquisition was set for 10 000 events per sample. The data were analyzed using FACSDiva Software version 4.1.2 (BD Biosciences).

### RNA isolation and reverse transcription

Total RNA was isolated using an RNeasy mini plus kit (Qiagen, Valencia, CA) according to the manufacturer's instructions. The concentration and quality of isolated RNA was determined using a ND-1000 Spectrophotometer (NanoDrop Technologies, Wilmington, DE). Complementary DNA (cDNA) was synthesized from 50 ng of total RNA using a Sensiscript Reverse Transcription Kit (Qiagen) according to the manufacturer's instructions.

### Quantitative RT-PCR

Both undifferentiated and differentiated hESCs and iPSCs in the culture plate were included in the quantitative RT-PCR analyses. Quantitative RT-PCR was performed for hESCs and iPSCs with Applied Biosystems Gene Expression Assays: *GAPDH* (Hs99999905_m1; 122-bp product), *DNMT3B* (Hs01003405_m1; 80-bp product), *TDGF1* (Hs02339496_g1; 102-bp product), *Nanog* (Hs2387400_g1; 109-bp product), *GDF3* (Hs00220998_m1; 65-bp product), *GABRB3* (Hs01115771_m1; 72-bp product), and *Oct4* (Hs00999632_g1; 77-bp product). All samples and the no-template controls were analyzed in triplicate. Quantitative RT-PCR was performed with an Applied Biosystems 7300 Real-Time PCR system using the following conditions: 40 cycles of 50°C, 2 min; 95°C, 10 min; and 95°C, 15 s; followed by 60°C, 1 min. The data were analyzed with a 7300 System SDS Software (Applied Biosystems). The cycle threshold (Ct) values were determined for every reaction. Relative quantification was calculated using the 2^−ΔΔCt^ method [42]. All data were normalized to the expression of the gene *GAPDH* whose expression does not change under the experimental conditions. The data are presented as mean fold-change. *Neural cells:* RNA isolation and reverse transcription of RT-PCR samples (≥10 spheres/sample) was performed as described above. The primer set for RT-PCR contained *Oct4*, *Nanog* for undifferentiated hESC; *α-fetoprotein* for endodermal lineage; *brachyury/T* for mesodermal lineage; *Musashi*, *Nestin*, and *PAX6* for neuroectodermal cells; *MAP-2*, *NF68* and *OTX2* for neuronal cells; *GFAP* for astrocytes, and *Olig1* for oligodentrocyte precursor cells. Data were normalized to the expression of the gene *GAPDH* whose expression does not change under the experimental conditions. Primer sequences are shown in supplementary Table S2. The RT-PCR reactions were performed in an Eppendorf Mastercycler using 35 PCR cycles with an initializing step at 95°C for 3 min, denaturation at 95°C for 30 s, annealing at 55°C for 30 s, and extension at 72°C for 1 min, followed by a final extension at 72°C for 5 min. The PCR products were analyzed by electrophoresis on a 1.5% agarose gel containing 0.4 µg/ml ethidium bromide (Sigma-Aldrich).

### Cell proliferation assay

Cell proliferation of the hESC lines was determined using a colorimetric immunoassay (Roche Diagnostics) based on the measurement of bromodeoxyuridine (BrdU) incorporation during DNA synthesis. The assay was performed according to the manufacturer's instructions. The hESC colonies at day 5 were labeled with BrdU-labeling solution overnight at 37°C. The hESCs (10^5^ cells/well) were added to a 96-well plate, with 8 replicates per cell line. Cells that were not labeled with BrdU were used as a background control. The absorbance of the samples was measured at 450 nm using a Viktor 1429 Multilabel Counter (PerkinElmer-Wallace, Norton, OH).

The cell viability and proliferation activity of the ASCs was assessed using PreMix WST-1 Cell Proliferation Assay System (Takara Bio Inc., Shiga, Japan). The ASCs (n = 7 donor cell samples, with 4 replicates per cell line) were seeded on a 48-well plate at a density of 4500 cells/cm^2^, and the proliferation was determined at 1, 4, 7 and 11 days. Briefly, the cell culture medium was removed and DPBS and PreMix WST-1 were added 10∶1. The well plate was incubated for 4 hours in 37°C and the cell proliferation activity was measured using a Viktor 1429 Multilabel Counter at 450 nm.

### Karyotyping

Karyotype analysis was performed by G-banding technique in Medix Laboratories Inc, Helsinki, Finland. In brief, the hESCs were treated with colchicine for 2 hours, and cells in metaphase were analyzed using conventional light microscopy (Olympus, BX 50). Karyotypes were determined using IKAROS-software designed for chromosome analysis (MetaSystems GmbH, Altlussheim, Germany). A total of 20 cells in metaphase were analyzed for each cell line.

### Analysis of pluripotency *in vitro*

The embryoid bodies (EBs) formed by mechanically dissecting the hESC and iPSC colonies were cultured without feeder cells in RegES or hES medium without bFGF for 4 weeks before RNA isolation. The media was changed every 2 to 3 days. RNA isolation and reverse transcription from EBs was performed as described above. The expression of markers characteristic of ectoderm (*PAX6* and *SOX1*, DNA Technologies Inc. Gaithersburg, MD), endoderm (*α-fetoprotein* and *SOX17*, DNA Technologies Inc.), and mesoderm (*α-cardiac actin*, Proligo, Sigma-Aldrich and *T*, DNA Technologies Inc.) development in EBs was determined using RT-PCR primers. *β-actin* (DNA Technologies Inc.) whose expression does not change under the experimental conditions was used as a control. Primer sequences are shown in supplementary Table S2. Negative controls contained sterilized water instead of the cDNA template. The RT-PCR reactions were performed in an Eppendorf Mastercycler as follows: denaturation at 95°C for 3 min and 40 cycles of denaturation at 95°C for 30 s, annealing at 57°C for 30 s, and extension at 72°C for 1 min, followed by a final extension at 72°C for 5 min. The PCR products were analyzed by electrophoresis on a 1.5% agarose gel containing 0.4 µg/ml ethidium bromide (Sigma-Aldrich) and 50 bp DNA standard (MassRulerTM DNA Ladder Mix, Fermentas).

### Analysis of pluripotency *in vivo*

Animal experiments with hESC lines Regea 06/015, Regea 06/040 and Regea 07/046 were performed at the infection-free animal facility of Karolinska University Hospital and experiments with hESC line Regea 08/013 at the animal facility of Turku Center for Disease Modeling and Department of Physiology, Institute of Biomedicine, University of Turku. Human ESCs were harvested from the culture plates using dispase or TrypleSelect and mechanical treatment. Five colonies (10^3^ to 10^4^ hESC) were washed twice in PBS and subsequently implanted beneath the testicular capsule of a young (6–8 week-old) severe combined immunodeficiency (scid)/beige male mouse (C.B.-17/GbmsTac-scid-bgDF N7, M&B, Ry, Denmark) or in the case of Regea 08/013 male Nude mouse (Hsd;Athymic Nude-Foxn1^nu^, Harlan). Three animals were used for cell line. Teratoma growth was determined by palpation once per week, and the mice were killed by cervical dislocation 8 to 9 weeks after implantation. The *in vivo* pluripotency of the hESC lines Regea 06/015, Regea 06/040 and Regea 07/046 was analyzed as previously described [8], [25]. Immunohistochemical studies of Regea 08013 cell line teratomas were done in paraffin sections. The dewaxed and rehydrated sections were treated in pressure cooker in 1 mM EDTA, pH 8, (Desmin and HNF3β) or 10 mM sodium citrate buffer, pH 6, (NCAM) for 5 minutes to reveal antigenic sites, cooled at room temperature (RT) for 2 hours, and rinsed in phosphate buffer saline (PBS). After rinsing with PBS the sections were incubated with primary antibodies (rabbit anti-desmin, sc-14026, mouse anti-HNF3β, sc-101060, purchased from Santa Cruz Biotechnology, or rabbit anti-NCAM, AB5032, purchased from Chemicon International. Primary antibodies were diluted (1∶1000, 1∶3000, and 1∶800 respectively) in PBS containing 0.05% Tween® and incubated overnight at +4 degrees. The next day, after several rinses with PBS, endogenous peroxidase was inactivated with 1% hydrogen peroxide in PBS for 20 minutes. After rinsing several times with PBS, the sections were incubated with secondary antibodies (HRP conjugated Dako Envision™+ Systems) for 30 minutes (RT). Slides were rinsed with PBS. Color was developed with diaminobenzidine substrate (Dako Envision™+ Systems). Sections were slightly counterstained with Mayer's hematoxylin, dehydrated, and mounted.

### Differentiation of hESCs to cardiomyocytes and neural cells

Cardiomyocyte differentiation was carried out as described previously [28] by co-culturing Regea 08/013 hESCs, maintained in RegES (p46) and in hES (p52) media, with mouse visceral endodermal-like (END-2) cells, which were a kind gift from Prof. Mummery, Humbrecht Institute, Utrecht, Netherlands. Briefly, undifferentiated hESC colonies were dissected mechanically into aggregates containing a few hundred cells and placed on the top of plated END-2 cells in hES culture medium without KO-SR and bFGF. Differentiating cell colonies were monitored by microscopy daily and the medium was changed after 5 days of culturing. After 12 days the 10% KO-SR (Invitrogen) was added to the medium. Differentiation was performed in 12-well plates, 15 hESC colony pieces in a well. The beating areas were dissociated by collagenase II treatment. The cells were plated on 0.1% gelatin coated cell culture plates in a medium containing 7.5% FBS.

Neural differentiation was performed as previously described [29]. Briefly, hESC (Regea 08/013) colonies maintained in RegES (p53) and in hES (p58) media, were mechanically dissociated into small clusters containing ∼3000 cells. Clusters were transferred into 6-well ultra low attachment plates (Nunc, Thermo Fisher Scientific, Rochester, NY) and cultured as floating aggregates, e.g. neurospheres, for up to 20 weeks in neural differentiation medium consisting of 1∶1 DMEM/F-12:Neurobasal media supplemented with 2 mM Glutamax, 1xB27, 1xN2 (all from Invitrogen), 25 U/ml penicillin-streptomycin (Lonza Group Ltd), and 20 ng/ml bFGF (R&D Systems). Medium was changed 3 times/week and the spheres were mechanically passaged weekly.

### Differentiation of ASCs to adipogenic, osteogenic and chondrogenic lineages

ASCs (passage 3) cultured in RegES medium were analyzed for their capacity to differentiate toward the adipogenic, osteogenic, and chondrogenic lineages with ASCs cultured in medium containing HS as reference. Briefly, osteogenic differentiation analyses were performed on ASCs seeded onto a 12-well plate at a density of 2.5×10^3^ cells/cm^2^ in RegES media supplemented with 150 µM L-ascorbic acid 2-phosphate (Sigma), 10 mM β-glycerophosphate (Sigma) and 100 nM dexamethasone (Sigma) in culture vessels were pre-coated with CELLstart. The control cultures were maintained in RegES media without supplements in pre-coated culture vessels. The cultures were maintained 14 days and osteogenic differentiation was detected by alkaline phosphatase activity staining.

Adipogenic differentiation was induced by culturing ASCs for two weeks in adipogenic media with an initial seeding density of 2×10^4^ cells/cm^2^ in culture vessels were pre-coated with CELLstart. For the adipogenic induction, RegES media supplemented with 33 µM biotin (Sigma), 1 µM dexamethasone, 100 nM insulin (Invitrogen) and 17 µM pantothenate (Fluka, Buchs, Switzerland) was used while the control cultures were maintained in RegES without adipogenic supplements. The cultures were analyzed using an Oil red O stain as an indicator of intracellular lipid accumulation.

The chondrogenic differentiation capacity analysis was assessed by micromass culture technique [1], [43]. Briefly, 1×10^5^ cells were seeded onto a 24-well culture plate in a 10 µl volume, and were let adhere for 3 h in a cell incubator prior to addition of the chondrogenic RegES media containing 1% ITS+1 (Sigma), 50 µM L-ascorbic acid 2-phosphate, 55 µM sodium pyruvate (Invitrogen), L-proline 23 µM (Sigma). The control cultures were maintained in RegES without chondrogenic supplements. The chondrogenic induction cultures and control cultures were performed without CELLstart pre-coating. The cultures were maintained for 14 days prior to analysis. Chondrogenesis was confirmed using Alcian blue stain.

### Statistical analyses

ANOVA by the methodology of mixed models was used for the proliferation data analysis of ASCs between the different culture media compositions within each separate experiment setup to account for the correlation of multiple measurements within donors. Mann-Whitney U test was applied in the surface marker expression of ASCs to compare the different culture conditions. The surface marker expression of ASCs was analyzed using Wilcoxon signed-rank statistics, which was used for pairwise comparison between the different culturing conditions. The statistical analyses were performed at the significance level p<0.05. The analyses were performed using SPSS software version 13 (SPSS Inc., Chicago, IL, USA).

## Supporting Information

Table S1 Complete Formulation for RegES Medium.
(0.05 MB DOC)

Table S2 Primer sequences.
(0.05 MB DOC)

Figure S1 Characterization of a hESC line Regea 06/015 derived in xeno-free medium RegES. A) Bright-field (scale bar, 500 µm) microscopic image of Regea 06/015 cell line at passage 3 after derivation. B) Bright-field (scale bar, 500 µm) microscopic image showing undifferentiated colony morphology of Regea 06/015 cell line at passage 35. C) RT-PCR analysis of in vitro-derived EBs of hESC line Regea 06/015 at passage 60 showing transcripts for AFP and SOX17 (endodermal markers), α-cardiac actin and T (Brachyuru; mesodermal markers), SOX1 and PAX6 (ectodermal markers), and β-actin as a housekeeping control. Lane 1, 50 bp DNA ladder. D) Fluorescent (scale bar, 200 µm) microscopic images showing undifferentiated colony morphology and qualitative immunocytochemistry of hESCs positive for the transcription factor Nanog, Oct4, Sox2, SSEA-4, and TRA-1-60. Insets represent DAPI staining.
(7.33 MB TIF)

Figure S2 Karyograms of hESC lines. A G-banding karyograms showing normal karyotypes of hESC lines, Regea 06/015 at passage 35, Regea 07/046 at passage 36, Regea 08/013 at passage 25 and Regea 06/040 at passage 71.
(9.41 MB TIF)

Figure S3 Histology of teratomas from cell line Regea 06/015 at passage 35. A) An overview of a teratoma. B) Endoderm: detail from the right part of the overview showing a lumen outlined by high cylindrical cells (*). The cells are compatible with endodermal differentiation of intestinal or respiratory type. C) Ectoderm: a cluster of neurons assembled into a ganglion like structure (arrow). Fibers tracts emanates from the cluster. D) Ectoderm: a squadmous epithelial island composed of fairly vacuolated cells (arrow). E) Mesoderm: lower part an island composed of hypertrophic chondrocytes (arrow).
(7.14 MB TIF)

Figure S4 Histology of teratomas from cell line Regea 07/046 at passage 17. A) This image shows tubular structures lined by cuboidal to cylindrical epithelium and aggregates consisting of smooth muscle cells (sm) are seen. B) Endoderm: some PASD+ cells (arrow) can be seen in the image, which are compatible with Goblet cells indicating the endodermal differentiation. C) Endoderm and mesoderm: a single tubule (*) lined by cuboidal to cylindrical epithelium. Note that it is embedded in a loosely arranged connective tissue - primitive mesenchyme. An interpretation would be that this represents endodermal (epithelial tubules) and mesodermal components. D) Endoderm or ectoderm: this image shows an epithelium lined tubule (*) embedded within a very loosely arranged mesenchyme. The epithelium can be interpreted as bilayered potentially representing a squamous variant. It is impossible to state if it is endodermal or ectodermal in origin.
(8.57 MB TIF)

Figure S5 Histology of teratomas from cell line Regea 08/013 at passage 86. A) Ectoderm: Large area of NCAM-positive, neuronal cells (arrow). B) Mesoderm: Bundles of muscle cells are stained positive for Desmin (arrow). C) Endoderm: Pseudostratified ciliated columnar epithelium stained positive for HNF3β (arrow).
(6.05 MB TIF)

Figure S6 Histology of teratomas from cell line Regea 06/040 at passage 34. A) and B) An overview of teratomas. Endoderm and mesoderm: note cartilage (c) and tubular structure (*) outlined by cylindrical cells suggesting mesodermal differentiation and varying amounts of Goblet cells suggesting endodermal differentiation. C) Endoderm: a high power view of an area from B is shown. Note smooth muscle like cells (sm) and cylindrical cells with scattered mucous producing Goblet cells. D) Ectoderm: a strong nuclear expression of MITF (arrow) specific for retinal pigment epithelial cells is seen indicating ectodermal differentiation.
(5.84 MB TIF)
