# Supplementary material for: A Defined and Xeno-Free Culture Method Enabling the Establishment of Clinical-Grade Human Embryonic, Induced Pluripotent and Adipose Stem Cells
Source: PLoS One. 2010 Apr 19;5(4):e10246. doi: 10.1371/journal.pone.0010246 (PMC2856688; doi:10.1371/journal.pone.0010246)
Supplement: Table S1 — Complete Formulation for RegES Medium. (0.05 MB DOC) [file pone.0010246.s001.doc]

**Supplementary table 1. Complete Formulation for RegES Medium**

| **Component** | **Concentration (mg/l)** | **Manufacturer** |
| --- | --- | --- |
| **Fatty acids *** |  |  |
| Linoleic acid | 1 | Cayman Chemicals |
| Arachidonic acid | 1 | Cayman Chemicals |
| Oleic acid | 1 | Sigma-Aldrich |
| **Amino acids** |  |  |
| Glycine | 53 | Sigma-Aldrich |
| L-histidine | 183 | Sigma-Aldrich |
| L-isoleucine | 615 | Sigma-Aldrich |
| L-methionine | 44 | Sigma-Aldrich |
| L-phenylalanine | 336 | Sigma-Aldrich |
| L-proline | 600 | Sigma-Aldrich |
| L-hydroxyproline | 15 | Sigma-Aldrich |
| L-serine | 162 | Sigma-Aldrich |
| L-threonine | 425 | Sigma-Aldrich |
| L-tryptophan | 82 | Sigma-Aldrich |
| L-tyrosine | 84 | Sigma-Aldrich |
| L-valine | 454 | Sigma-Aldrich |
| **Vitamins** |  |  |
| Thiamine | 9 | Sigma-Aldrich |
| Retinol * | 0.5 | Sigma-Aldrich |
| **Antioxidants** |  |  |
| Glutathione | 1,5 | Sigma-Aldrich |
| Ascorbic acid | 50 | Sigma-Aldrich |
| **Proteins** |  |  |
| Human serum albumin | 10000 | Sigma-Aldrich |
| Insulin | 10 | Invitrogen |
| Transferrin | 8 | Sigma-Aldrich |
| FGF basic | 0.008 | R&D Systems |
| Activin A * | 0.005 | R&D Systems |
| **Trace elements** |  |  |
| Trace elements B | 1:1000 | Cellgro |
| Trace elements C | 1:1000 | Cellgro |
| Selenium | 0.00001 | Sigma-Aldrich |
| **Other components** |  |  |
| NEAA | 1% | Cambrex Bio Science |
| L-glutamine | 2 mM | Invitrogen |
| β-mercaptoethanol | 0.1 mM | Invitrogen |

Basal medium: KnockOut DMEM (Invitrogen)

* Components added after optimization
