# Supplementary material for: A Defined and Xeno-Free Culture Method Enabling the Establishment of Clinical-Grade Human Embryonic, Induced Pluripotent and Adipose Stem Cells
Source: PLoS One. 2010 Apr 19;5(4):e10246. doi: 10.1371/journal.pone.0010246 (PMC2856688; doi:10.1371/journal.pone.0010246)
Supplement: Table S2 — Primer sequences. (0.05 MB DOC) [file pone.0010246.s002.doc]

Supplementary table 2. Primer sequence list.

| **Primer** |  | **Primer sequence** |  |  | **Size (bp)** |
| --- | --- | --- | --- | --- | --- |
| Oct4  Nanog |  | *F* 5´ CGTGAAGCTGGAGAAGGAGAAGCTG 3´  *R 5´* AAGGGCCGCAGCTTACACATGTTC 3´  F5´ TGCAAATGTCTTCTGCTGAGAT 3’  R5’ GTTCAGGATGTTGGAGAGTTC 3’ |  |  | 245  286 |
| AFP |  | *F* 5´ GCTGGATTGTCTGCAGGATGGGGAA 3´  *R* 5´ TCCCCTGAAGAAAATTGGTTAAAAT 3´ |  |  | 216 |
| Brachyury/T |  | *F* 5´ GCTTCAAGGAGCTCACCAAT 3´  *R* 5´ CACCGCTATGAACTGGGTCT 3´ |  |  | 425 |
| Musashi |  | *F* 5´ AGCTTCCCTCTCCCTCATTC 3´  *R* 5´ GAGACACCGGAGGATGGTAA 3´ |  |  | 161 |
| Nestin |  | F 5´ CAGCTGGCGCACCTCAAGATG 3´  R 5´ AGGGAAGTTGGGCTCAGGACTGG |  |  | 208 |
| PAX6  OTX2 |  | *F* 5´ AACAGACACAGCCCTCACAAACA 3´  *R* 5´ CGGGAACTTGAACTGGAACTGAC 3´  F5’ CGCCTTACGCAGTCAATGGG 3’  R5’ CGGGAAGCTGGTGATGCATAG 3’ |  |  | 174  641 |
| MAP-2 |  | *F* 5´ AATAGACCTAAGCCATGTGACATCC 3´  *R* 5´ AGAACCAACTTTAGCTTGGGCC 3´ |  |  | 132 |
| NF68 |  | *F* 5´ GAGTGAAATGGCACGATACCTA 3´  *R* 5´ TTTCCTCTCCTTCTTCACCTTC 3´ |  |  | 473 |
| GFAP |  | *F* 5´ GCTCGATCAACTCACCGCCAACA 3´  *R* 5´ GGGCAGCAGCGTCTGTCAGGTC 3´ |  |  | 207 |
| Olig1 |  | *F* 5´ TTGCATCCAGTGTTCCCGATTTAC 3´  *R* 5´ TGCCAGTTAAATTCGGCTACTACC 3´ |  |  | 389 |
| GAPDH  Β-Actin  Α-cardiactin  AFP  SOX17  SOX1 |  | *F* 5´ AGCCACATCGCTCAGACACC 3´  *R* 5´ GTACTCAGCGGCCAGCATCG 3´  F5’ GTC TTC CCC TCC ATC GTG 3’  R5’ GGG GTG TTG AAG GTC TCA AA 3’  F5’ GGA GTT ATG GTG GGT ATG GGT C 3’  R5’ AGT GGT GAC AAA GGA GTA GCC A 3’  F5’ AGA ACC TGT CAC AAG CTG TG 3’  R5’ GAC AGC AAG CTG AGG ATG TC 3’  F5’ CGC ACG GAA TTT GAA CAG TA 3’  R5’ CAC ACG TCA GGA TAG TTG CAG 3’  F5’ AAA GTC AAA ACG AGG CGA GA 3’  R5’ AAG TGC TTG GAC CTG CCT TA 3’ |  |  | 302  302  486  672  166  158 |
