# Supplementary figures and images for: A Defined and Xeno-Free Culture Method Enabling the Establishment of Clinical-Grade Human Embryonic, Induced Pluripotent and Adipose Stem Cells
Source: PLoS One. 2010 Apr 19;5(4):e10246. doi: 10.1371/journal.pone.0010246 (PMC2856688; doi:10.1371/journal.pone.0010246)

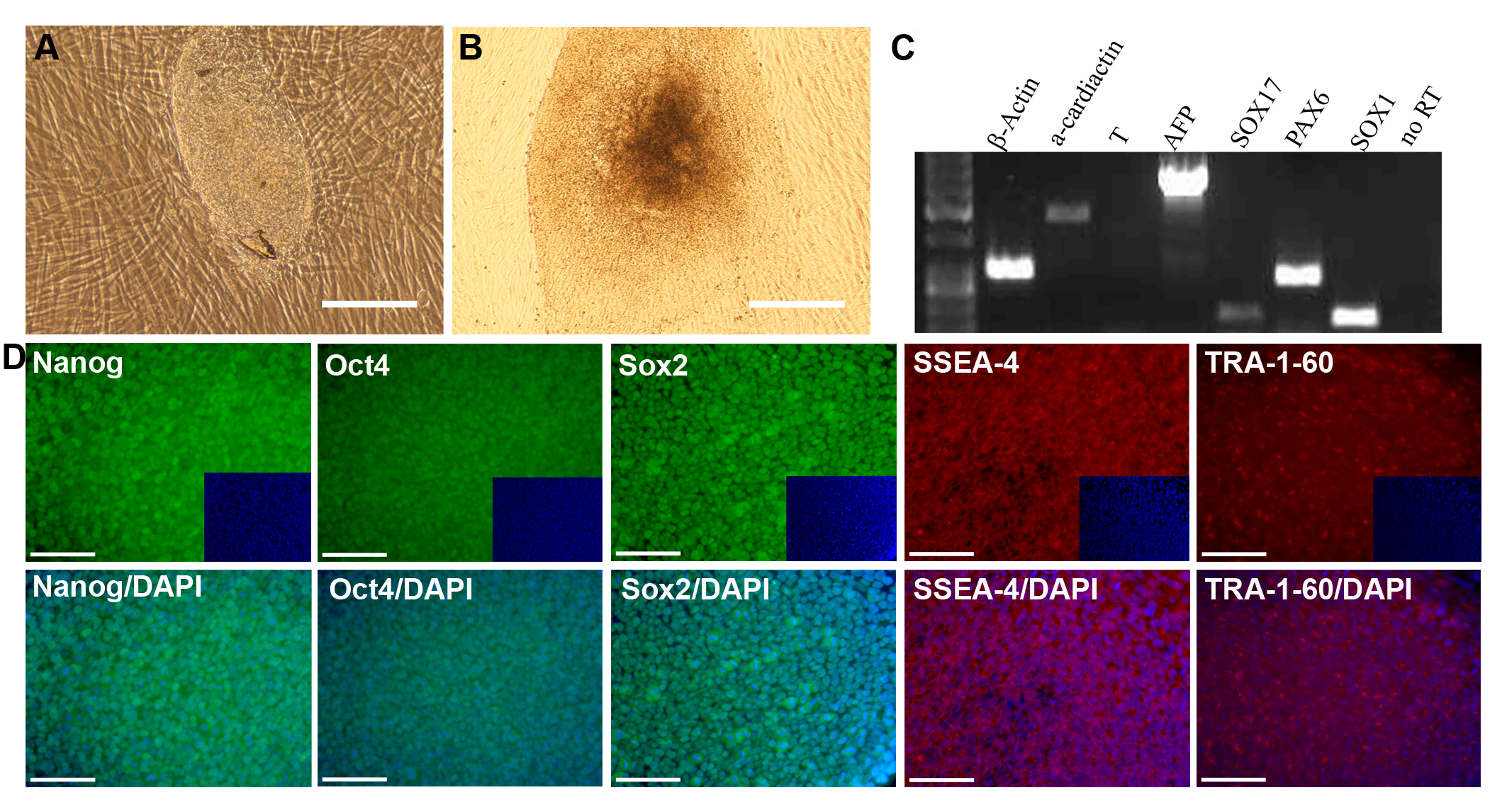

Supplement: Figure S1 — Characterization of a hESC line Regea 06/015 derived in xeno-free medium RegES. A) Bright-field (scale bar, 500 µm) microscopic image of Regea 06/015 cell line at passage 3 after derivation. B) Bright-field (scale bar, 500 µm) microscopic image showing undifferentiated colony morphology of Regea 06/015 cell line at passage 35. C) RT-PCR analysis of in vitro-derived EBs of hESC line Regea 06/015 at passage 60 showing transcripts for AFP and SOX17 (endodermal markers), α-cardiac actin and T (Brachyuru; mesodermal markers), SOX1 and PAX6 (ectodermal markers), and β-actin as a housekeeping control. Lane 1, 50 bp DNA ladder. D) Fluorescent (scale bar, 200 µm) microscopic images showing undifferentiated colony morphology and qualitative immunocytochemistry of hESCs positive for the transcription factor Nanog, Oct4, Sox2, SSEA-4, and TRA-1-60. Insets represent DAPI staining. (7.33 MB TIF) [file pone.0010246.s003.tif]

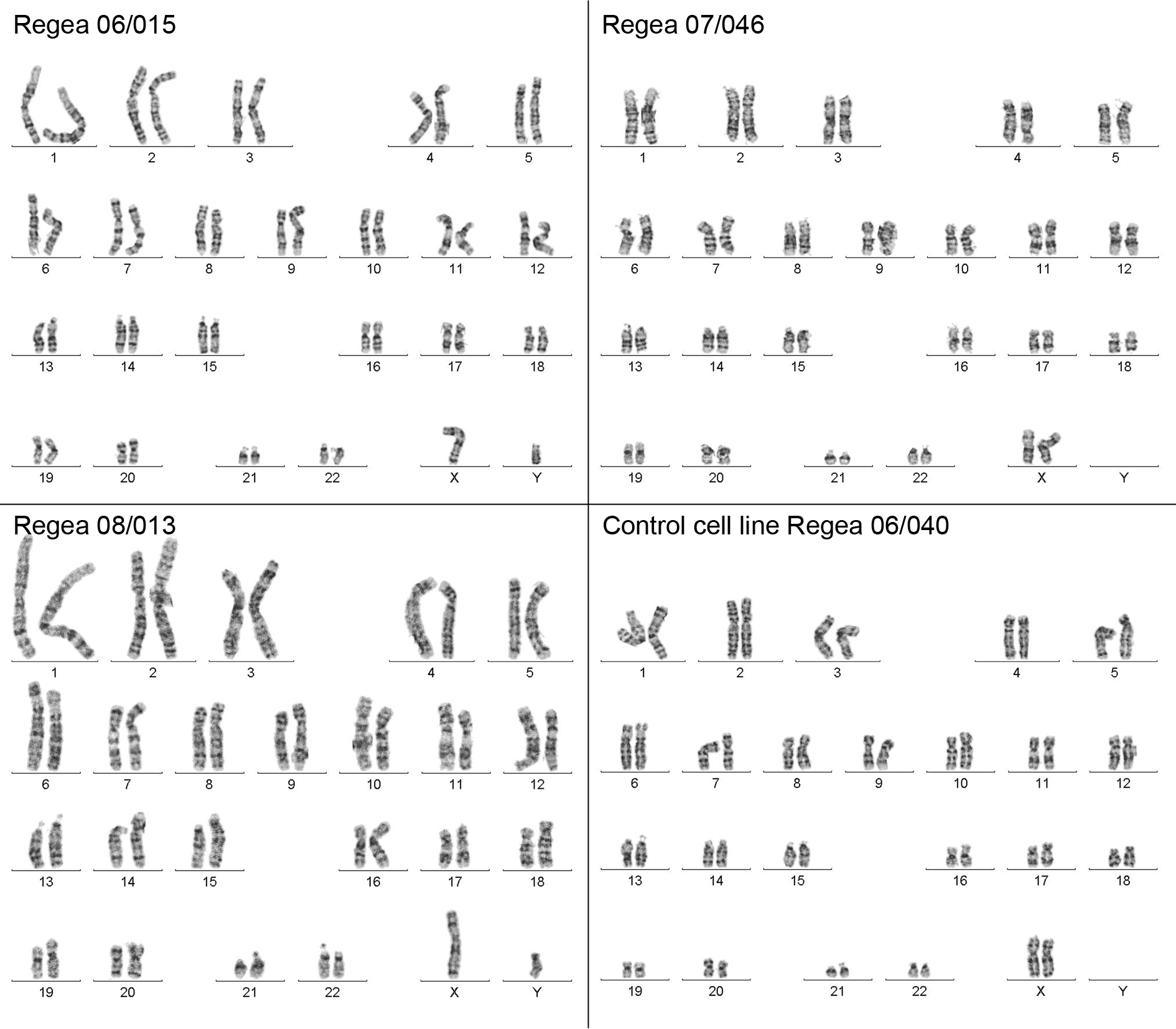

Supplement: Figure S2 — Karyograms of hESC lines. A G-banding karyograms showing normal karyotypes of hESC lines, Regea 06/015 at passage 35, Regea 07/046 at passage 36, Regea 08/013 at passage 25 and Regea 06/040 at passage 71. (9.41 MB TIF) [file pone.0010246.s004.tif]

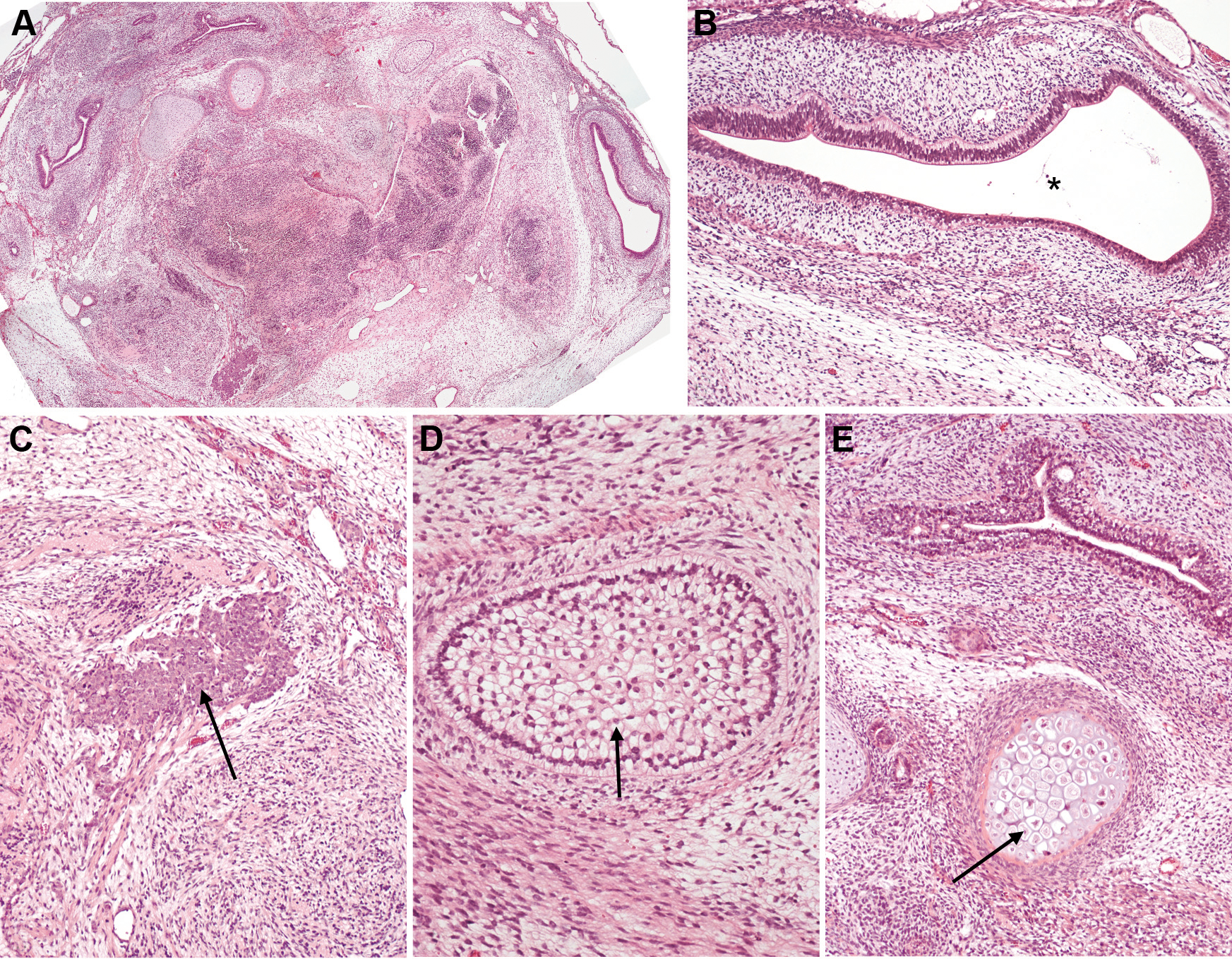

Supplement: Figure S3 — Histology of teratomas from cell line Regea 06/015 at passage 35. A) An overview of a teratoma. B) Endoderm: detail from the right part of the overview showing a lumen outlined by high cylindrical cells (*). The cells are compatible with endodermal differentiation of intestinal or respiratory type. C) Ectoderm: a cluster of neurons assembled into a ganglion like structure (arrow). Fibers tracts emanates from the cluster. D) Ectoderm: a squadmous epithelial island composed of fairly vacuolated cells (arrow). E) Mesoderm: lower part an island composed of hypertrophic chondrocytes (arrow). (7.14 MB TIF) [file pone.0010246.s005.tif]

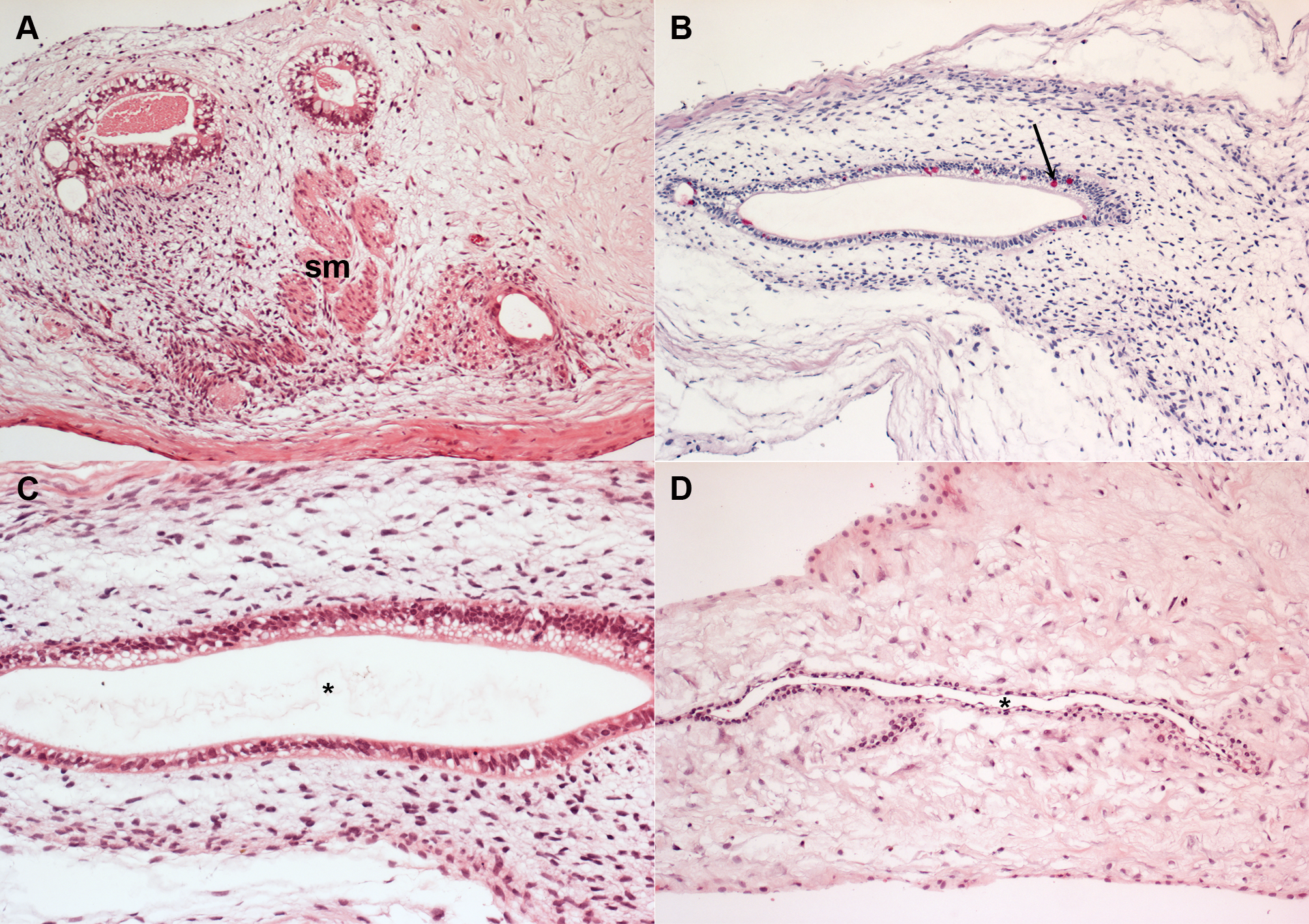

Supplement: Figure S4 — Histology of teratomas from cell line Regea 07/046 at passage 17. A) This image shows tubular structures lined by cuboidal to cylindrical epithelium and aggregates consisting of smooth muscle cells (sm) are seen. B) Endoderm: some PASD+ cells (arrow) can be seen in the image, which are compatible with Goblet cells indicating the endodermal differentiation. C) Endoderm and mesoderm: a single tubule (*) lined by cuboidal to cylindrical epithelium. Note that it is embedded in a loosely arranged connective tissue - primitive mesenchyme. An interpretation would be that this represents endodermal (epithelial tubules) and mesodermal components. D) Endoderm or ectoderm: this image shows an epithelium lined tubule (*) embedded within a very loosely arranged mesenchyme. The epithelium can be interpreted as bilayered potentially representing a squamous variant. It is impossible to state if it is endodermal or ectodermal in origin. (8.57 MB TIF) [file pone.0010246.s006.tif]

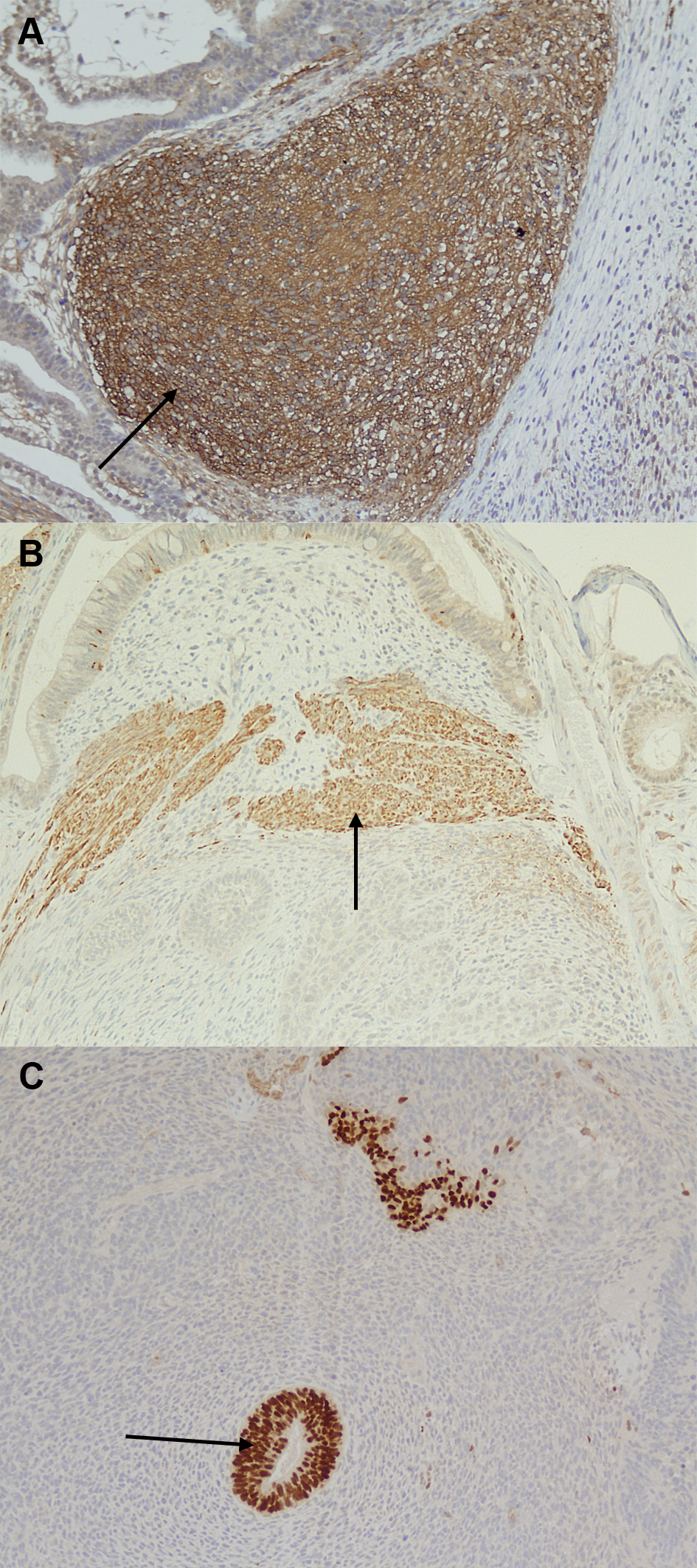

Supplement: Figure S5 — Histology of teratomas from cell line Regea 08/013 at passage 86. A) Ectoderm: Large area of NCAM-positive, neuronal cells (arrow). B) Mesoderm: Bundles of muscle cells are stained positive for Desmin (arrow). C) Endoderm: Pseudostratified ciliated columnar epithelium stained positive for HNF3β (arrow). (6.05 MB TIF) [file pone.0010246.s007.tif]

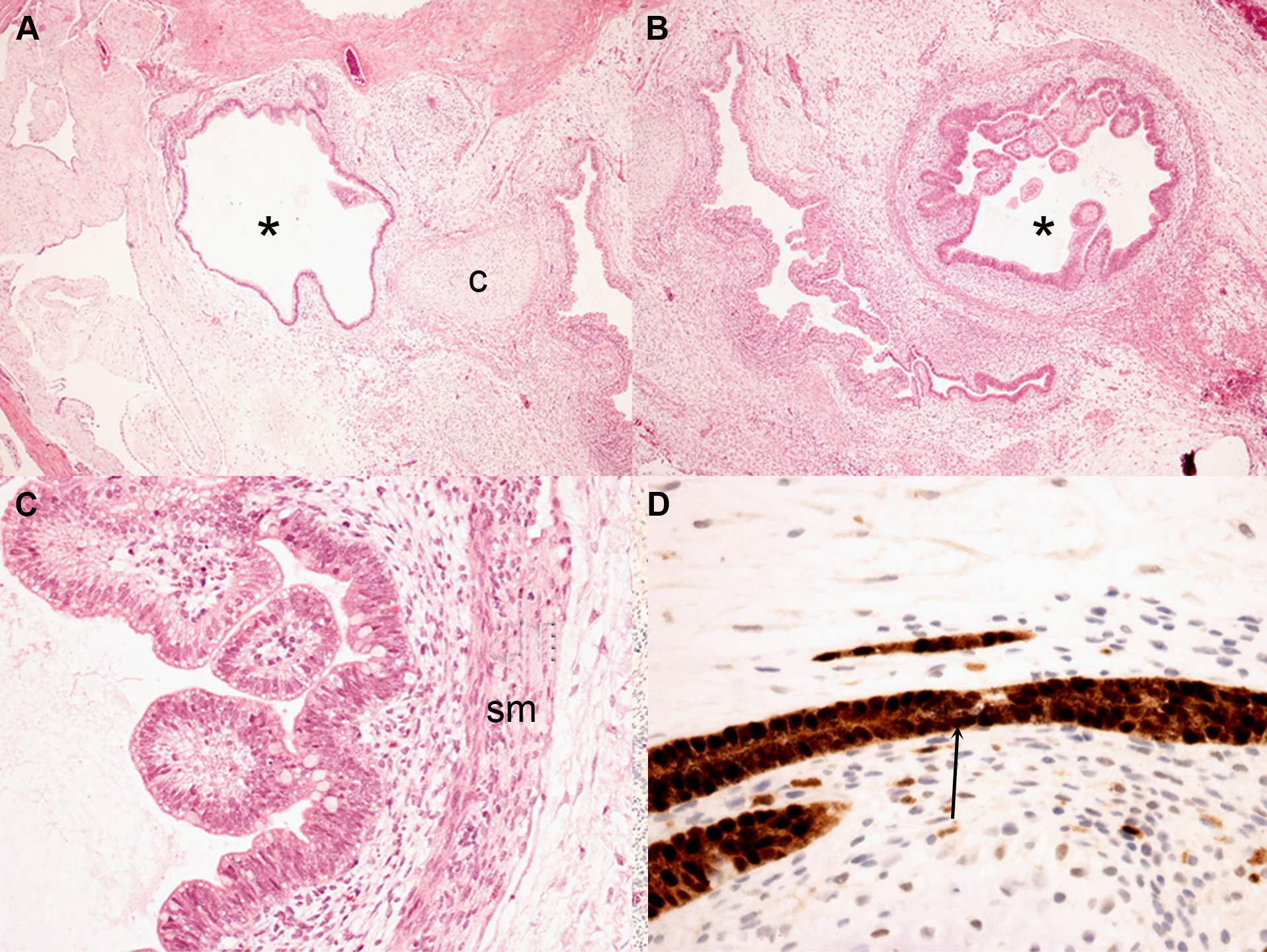

Supplement: Figure S6 — Histology of teratomas from cell line Regea 06/040 at passage 34. A) and B) An overview of teratomas. Endoderm and mesoderm: note cartilage (c) and tubular structure (*) outlined by cylindrical cells suggesting mesodermal differentiation and varying amounts of Goblet cells suggesting endodermal differentiation. C) Endoderm: a high power view of an area from B is shown. Note smooth muscle like cells (sm) and cylindrical cells with scattered mucous producing Goblet cells. D) Ectoderm: a strong nuclear expression of MITF (arrow) specific for retinal pigment epithelial cells is seen indicating ectodermal differentiation. (5.84 MB TIF) [file pone.0010246.s008.tif]
